# Comparison of Percutaneous Kyphoplasty with or without Pedicle Screw Fixation in Osteoporotic Thoracolumbar Vertebral Fractures: A Retrospective Study

**DOI:** 10.1155/2021/4745853

**Published:** 2021-06-29

**Authors:** Dichao Huang, Jichong Ying, Dingli Xu, Jianming Chen, Jianlei Liu, Tianming Yu, Yunqiang Zhuang, Long Zhou

**Affiliations:** Ningbo No.6 Hospital, Zhejiang, Ningbo 315000, China

## Abstract

**Background:**

Osteoporotic thoracolumbar compression fractures have become a great social burden due to the aging tendency of population. This study is aimed at comparing the clinical and radiological outcomes of percutaneous kyphoplasty with or without pedicle screw fixation in patients with osteoporotic thoracolumbar fractures. *Hypothesis*. There is a difference in clinical outcomes between percutaneous kyphoplasty with pedicle screw fixation and percutaneous kyphoplasty.

**Methods:**

This retrospective study included 87 patients who received percutaneous kyphoplasty with or without pedicle screw fixation between October 2015 and October 2017 at Ningbo No.6 Hospital and were followed for 2 years. A total of 40 patients received percutaneous kyphoplasty with pedicle screw fixation (PKPF group), and the other 47 patients had percutaneous kyphoplasty only (PKP group). The outcomes were measured using the visual analogue scale (VAS), Oswestry Disability Index (ODI), Cobb angle (CA), and anterior vertebra height rate (AVHr), which were calculated at preoperative admission and each follow-up visit. Complications including postoperative back pain, refracture, and fixation failure were collected from medical records.

**Results:**

There was no significant difference in baseline characteristics or preoperative data between the two groups (*p* < 0.05) but significantly better improvements in VAS, ODI, CA, and AVHr at 12- and 24-month follow-up visits in the PKPF group compared with those of the PKP group. 23 (48.9%) patients in the PKP group had complications, whereas only 5 (12.5%) patients in the PKPF group presented complications including 2 postoperative back pain and 1 fixation failure (*p* = 0.04).

**Conclusions:**

PKPF obtained longer correction and better improvement in VAS, ODI, and CA in patients with osteoporotic thoracolumbar vertebral fractures than PKP.

## 1. Background

Osteoporosis vertebral compression fracture (OVCF) is prevailing with the aging of population. With around 1.4 million new cases every year, it has become a great social, health, and economic burden since patients will be unable to perform daily activities due to severe back pain [1]. Over the past decades, percutaneous kyphoplasty (PKP) has been widely used for the treatment of osteoporosis and vertebral compression fractures, because it is a minimal invasive surgery to achieve many benefits on short-term prognosis including pain relief, shortened hospital stay, and restoration of vertebral body height [2]. Hu et al. reported that after percutaneous balloon kyphoplasty, 91 patients with osteoporotic vertebral compression fracture achieved satisfactory improvement in visual analogue scale (VAS), Cobb angle (CA), and anterior vertebra height rate (AVHr) compared with those preoperative indices (*p* < 0.05) [3].

However, some researchers found the disadvantages of PKP including kyphosis, refracture, back pain, infection, and adjacent vertebral fracture [4]. Li et al. reported that 30 out of 230 patients who received PKP with bilateral approach had recollapse during follow-up visits, and the possible reasons were low bone mineral density and low volume of injected cement [5]. To minimize the postoperative complications of OVCF, pedicle screw fixation combined with percutaneous kyphoplasty (PKPF) is prevailing, because PKPF can decrease vertebral refracture, adjacent vertebral fracture, and kyphosis [6]. Korovessis et al. retrospectively collected clinical and radiological data of 36 patients treated with percutaneous short fixation plus kyphoplasty and documented that this surgical method significantly reduced spinal deformity and pain with few complications [7], whereas there was no comparison of percutaneous kyphoplasty with or without pedicle screw fixation in the treatment of single segment osteoporosis vertebral compression fracture on follow-up. Our present study is aimed at comparing the clinical outcomes and complications of osteoporosis vertebral compression fracture patients who received kyphoplasty with or without pedicle screw fixation.

## 2. Patients and Methods

**Figure 1:**
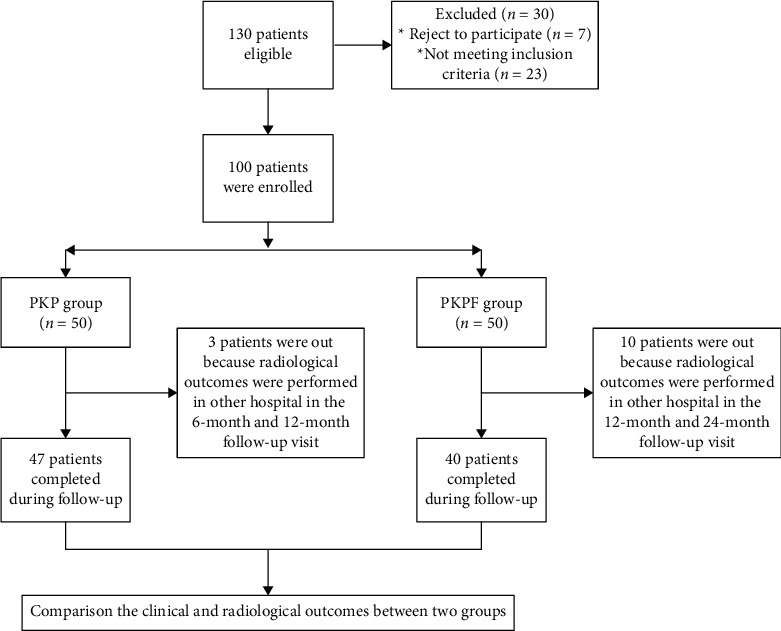
Flow of participants through in this study.

This is a retrospective study, and all data were retrieved from Ningbo No.6 Hospital. All enrolled cases met the following inclusion criteria: (1) patients aged > 55 years old without trauma history, previous lumbar fracture, or thoracolumbar surgery; (2) diagnosed as T11-L2 single segment thoracolumbar osteoporotic vertebral compression fracture by MRI and CT scan (defined as vertebral height loss > 25%) [8]; and (3) bone mineral density less than -2.5 standard deviation (SD) of normal. The exclusion criteria were as follows: (1) without intact pedicle or posterior wall of the fractured vertebral; (2) more than two-segment vertebrae fractured, pathological fracture, or other diseases which might affect clinical outcomes including serious cardiovascular disease, mental disorder, or uremia; and (3) without complete follow-up data. A total of 87 qualified patients who received percutaneous kyphoplasty with or without pedicle screw fixation in Ningbo No.6 Hospital from October 2015 and October 2017 and had 2 years followed up visit were enrolled for analysis, including 47 patients treated with percutaneous kyphoplasty (the PKP group) and 40 patients treated with percutaneous kyphoplasty with pedicle screw fixation (the PKPF group), shown in Figure 1. The clinical outcomes, radiological, and demographic data were collected from medical records, and there was no difference between the two groups in rehabilitation protocols. Institutional ethical approval was obtained before data collection. All patients signed informed consent for unnamed involvement for research purposes at admission.

### 2.1. Surgery Procedure

In the PKP group, all procedures were performed under local anesthesia in the prone position; then, the fractured vertebra was located by C-arm fluoroscopy. A cannula was placed percutaneously into the vertebral body through a bilateral pedicles, which allowed the placement of two inflatable balloons. The position of the cannula was identified by intraoperative X-rays. After that, the balloons were inflated to compact the surrounding trabecular bone and create an enclosed cavity filled with PK and PMMA bone cement.

**Figure 2:**
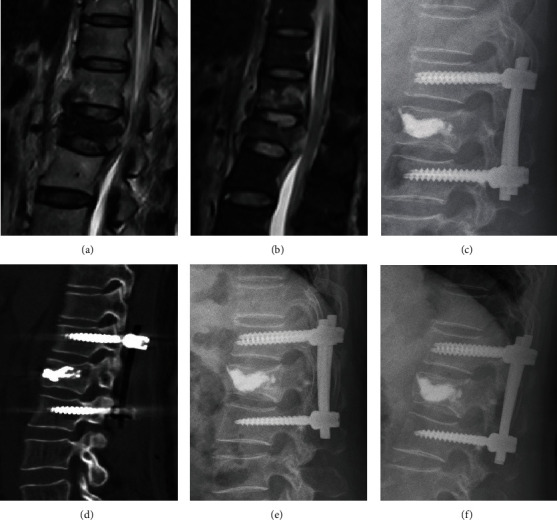
A female patient aged 62 years old, L1 OVCF caused by fall. (a, b) Postoperative magnetic resonance imaging showed L1 vertebral compression fracture. (c, d) Immediately postoperative CT scans and lateral X-ray showed that the fractured vertebra was filled with bone cement, and pedicle screws were placed into the adjacent vertebral. (e, f) Lateral X-rays at 6-month and 2-year follow-up visit indicated that satisfying correction and no recollapsed or refracture.

In the PKPF group, all patients received endotracheal anesthesia. Procedures were monitored under biplane fluoroscopy and continuous neuromonitoring during operation. The fractured vertebra was augmented with PK and PMMA bone cement as aforementioned. The adjacents above and below the intact vertebrae were located with four targeting cannulated needles at each pedicle, and all additional instruments were arranged by K-wires, which passed through each cannulated needle. Then, the multiaxial cannulated pedicle screws (Sanyou, Shanghaisanyou, China) were inserted by the extender sleeves into the pedicles of adjacent vertebras above and below the injured vertebra. Finally, two appropriate length and preflexed rods were placed through the minimal stab wound, and the position was controlled by C-arm fluoroscopy (Figure 2). Both groups retained a brace for 3 months and oral nonsteroid anti-inflammatory drugs accordingly, and all patients were oral alendronate sodium to antiosteoporosis after they were diagnosed osteoporosis.

### 2.2. Outcome Assessments

Clinical outcomes include VAS and Oswestry Disability Index (ODI). The VAS (from 0, no pain, to 10, worst pain) was used to measure the back pain at preoperative admission and each follow-up visit [9]. And the ODI scores consisting of 10 questions were used to evaluate functional capacity, indicating a worse prognosis with higher percentage [10]. The ODI score is a prevailing method of high reproducible and reliable measurement in patients with lower back pain.

The immediate postsurgical anteroposterior and lateral X-rays were used to assess the reduction of the injured vertebra, bone cement distribution, and position of implements. The CA and AVHr were measured under lateral lumbar X-rays at preoperative admission and each follow-up visit, which were used to evaluate the thoracolumbar alignment [11], and all data were measured by two radiological doctors. The definition of recollapse of the operated vertebra is a ≥4 mm decrease of vertebral body height compared with that in immediately postoperative lateral X-rays [12]. Complications including wound infection, postoperative kyphosis, refracture, and implement failure were collected from medical records by two independent orthopedic surgeons.

### 2.3. Statistics

Continuous data were shown as mean ± SD. The baseline characteristics were analyzed by *χ*^2^ test or independent *t*-test. The outcomes of preoperative and postoperative follow-up visits in each group were analyzed using the paired *t*-test, and the independent *t*-test was used to compare the difference between the two groups at preoperative and each follow-up visit. Statistical analyses were performed by SPSS for Windows version 22 (SPSS Inc., Chicago, USA). And the survival analysis was used for the comparison of complications between the two groups. A two-tailed *p* value less than 0.05 was considered statistically significant.

## 3. Results

A total of 87 OVCF patients treated with either operation were enrolled in this retrospective study, including 47 patients treated with PKP and 40 patients treated with PKPF. There was no significant difference in the baseline characteristics between the two groups (*p* < 0.05), such as age, gender, body mass index, and fractured level (Table 1), whereas the hospital stay was significantly shorter in the PKP group than in the PKPF group (3.5 ± 1.4 vs. 6.9 ± 2.1, *p* < 0.05).

As for the clinical outcomes (Table 2), there were significantly longer operation times and more operation blood loss in the PKPF group compared with those in the PKP group (8.6 ± 2.1 ml and 46.8 ± 9.7 min vs. 74.7 ± 9.3 ml and 81.7 ± 12.7 min, respectively, *p* < 0.05 for both). There was a significantly better decrease in VAS at 1- and 6-month follow-up visit in the PKPF group compared with that in the PKP group (*p* < 0.05), whereas there was no significant difference between the two groups at 12- and 24-month follow-up visit (*p* > 0.05). Significant improvement in ODI was found in the two groups at 1-, 6-, and 12-month follow-up visits (*p* < 0.05), and there was significantly improvement of ODI in the PKPF groups at 12- and 24-month follow-up visits (9.1 ± 3.4 and 7.4 ± 2.1 vs. 13.7 ± 5.2 and 12.4 ± 3.5, respectively, *p* < 0.05 for both).

In all patients, the X-rays and CT scan at preoperative admission and each follow-up visit were used to analyze the radiological paraments including CA and AVHr (Table 3). All patients achieved satisfactory recovery in AVHr at 1-month follow-up visit compared with that at preoperative admission in both groups (*p* < 0.05), whereas the AVHr was significantly lower at 12-month follow-up visit in the PKP group (81.7 ± 3.2 vs. 91.3 ± 1.5, *p* < 0.05). There were significantly higher AVHr in the PKPF group than the PKP group at each follow-up visit (*p* < 0.05), and the PKPF group showed longer improvement maintaining than the PKP group. As for CA, both groups yielded satisfactory recovery after operation (6.4 ± 3.1° and 3.1 ± 2.5° vs. 20.5 ± 2.5° and 21.1 ± 3.1°, *p* < 0.05), and there were significantly better recoveries at each follow-up visit in the PKPF group than in the PKP group. There was no significant difference in CA between each follow-up visit in the PKPF group. However, the CA was significantly increased at 6-month follow-up visit compared with that at 1-month follow-up in the PKP group (9.7 ± 4.5 vs. 6.4 ± 3.1, *p* < 0.05).

**Figure 3:**
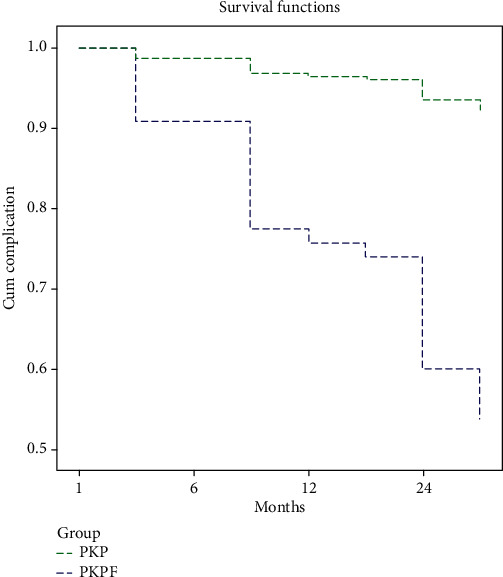
The survival analysis of complications between two groups.

In the PKP group, a total of 23 (48.9%) patients had complications, including cement leakage (*n* = 10), fractured vertebra recollapse (*n* = 12), and reoperation due to refracture (*n* = 2), and there were significantly fewer complications in the PKPF group including cement leakage (*n* = 2), wound infection (*n* = 1), and recollapse at final follow-up visit (*n* = 2, *p* < 0.05). The survival analysis (Figure 3) showed that the surgery method was an independent factor affecting osteoporotic thoracolumbar compression fractures (*p* < 0.001). Compared with PKP, patients receiving PKPF had a lower risk of complications, HR = 7.74 (95% CI: 2.812~ 21.298).

## 4. Discussion

Nowadays, the incidence of osteoporotic vertebra fracture is trending upwards in an aged society, creating considerable social and economic burdens and decreasing patient living quality. Many patients may have kyphosis and other comorbidities including pneumonia and thrombosis without proper treatments [13]. The most commonly used operation is PKP, which has achieved satisfactory outcomes evaluated by VAS, ODI, and local CA at each follow-up visit compared with those at preoperative admission (*p* < 0.05) [14]. However, with the accumulation of clinical cases and prolonged follow-up period, some disadvantages were documented including cement leakage, recollapse, infection, and even refracture [15, 16]. Wei et al. reported that 1 (5%) patient had an adjacent vertebral fracture after PKP [17], and Wang et al. found that 79 (38.9%) patients who were treated with PKP had recollapse during followed up visits [18].

To minimize complications and maintain stronger support to vertebras, Pingel et al. developed PKPF for the treatment of OVCF [19], and some publications showed that PKPF achieved satisfactory clinical outcomes. Wu et al. reported that a total of 36 patients with osteoporotic single segment vertebral fracture received percutaneous kyphoplasty combined with posterior pedicle screw-rod fixation. All patients achieved significant recovery in VAS compared with those at preoperative admission, and there was no recollapse up to the final followed up visit [20]. Similarly, Elmasry et al. reported a finite element study about the comparison of percutaneous kyphoplasty with or without pedicle screws and found that PKP had larger range of motion (ROM) than PKPF, but PKPF showed a higher level of support to vertebras [21].

In this study, VAS, ODI, AVHr, and CA were all significantly improved in the PKPF group than those in the PKP group at follow-up visits. Although there were significant improvements in vertebral body height, CA, and VAS in both two groups compared with those of the preoperative data, patients treated with PKPF yielded significantly better improvements than those with PKP [22]. As for radiological outcomes, all patients have achieved restoration at 1-month followed up visit compared with preoperative conditions, but there were significantly worse findings in AVHr at 6-month followed up visit than that at 1 month in patients treated with PKP. In contrast, there was significantly better improvement in AVHr and CA at each followed up visit in the PKPF group than those in the PKP group, with longer correction maintained. Li et al. reported the same outcomes that all of the 50 patients with OVCF treated with PKP had yielded initial improvement in VAS, ODI, vertebral body height, and kyphosis angle, but significant loss of correction in vertebral body height and kyphosis angle at the final follow-up visit, with VAS score and ODI showing similarly patterns [23]. The possible reason maybe that PKP can initially restore the height of the vertebral body and local kyphosis, but there is a subsequence height loss due to intravertebral cleft [24] and osteoporosis [25]. The areas of intravertebral cleft consisted with necrotic cancellous bone, hyaline cartilage, and fractured callus that commonly associated with avascular necrosis [26]. That may induce instability, back pain, and recollapse, so patients may show the loss of vertebral body height, CA, and recurrence of back pain without strong support provided by pedicle screw fixation.

Other researches had documented similarly results. Lee et al. reported that 31 patients with OVCF received PKP, and all of them achieved significant postoperative improvements in the clinical and radiological findings during early follow-up, but 26 (78.8%) presented vertebral cement leakage, 5 (15.2%) were recollapse, and 6 (18.2%) had refracture [27]. Although the reason of refracture is still controversial, many studies reported the possible reason maybe that cement leakage into disk [28, 29].

Although there were many studies about comparison of the two procedures, this study is the first to show PKPF can achieve a longer correction period and stronger support of the vertebra 1 year after surgery in OVCF patients than PKP. Second, all cases were performed by a single experienced spine surgeon in our hospital with the same protocol; thus, the procedure has been highly consistent and comparable. Some limitations include a retrospective study design with a relatively small number of cases, and whether patients had used antiosteoporosis medication was not taken into account [30–33].

In conclusion, PKPF for patients with osteoporotic thoracolumbar fractures can not only achieve favorable outcomes but also maintain longer correction and stronger support of the vertebra than PKP. However, more randomized controlled trials are still needed to confirm these findings.

## Figures and Tables

**Table 1:** The baseline characteristics of patients in the two groups.

Variable	PKP group (*n* = 47)	PKPF group (*n* = 40)	*p* value
Age (years)	65.1 ± 13.8	63.3 ± 14.9	0.53
Body mass index	22.7 ± 2.1	22.1 ± 1.9	
Gender			
Male	17	13	0.45
Female	30	27	
Fracture cause, *n*			
Fall	35	31	0.47
Vehicle injure	12	9	
Fracture vertebra			
T11	10	7	0.89
T12	12	11	
L1	17	13	
L2	8	9	
Hospital stay (days)	3.5 ± 1.4	6.9 ± 2.1	0.03^∗^

^∗^ *p* < 0.05.

**Table 2:** Comparison of clinical outcomes between the two groups.

Variable	PKP group (*n* = 47)		PKPF group (*n* = 40)		*p*_1_
VAS		*p*_2_		*p*_2_	
Preoperative	6.9 ± 2.7		6.5 ± 2.2		0.58
1 month	2.8 ± 1.6	<0.001	2.1 ± 0.8	<0.001	0.03^∗^
6 months	1.7 ± 1.1	0.04	1.0 ± 0.4	0.03	0.04^∗^
12 months	1.3 ± 0.5	0.45	0.7 ± 0.5	0.63	0.35
24 months	0.9 ± 0.7	0.52	0.6 ± 0.4	0.45	0.19
ODI					
Preoperative	68.3 ± 9.7		65.8 ± 8.4		0.75
1 month	27.3 ± 5.9	<0.001	23.4 ± 6.7	<0.001	0.51
6 months	19.3 ± 4.2	0.04	15.7 ± 48	0.02	0.16
12 months	13.7 ± 5.2	0.03	9.1 ± 3.4	0.04	0.03^∗^
24 months	12.4 ± 3.5	0.24	7.4 ± 2.1	0.14	0.04^∗^
Complications					
Cement leakage	9		2		<0.001
Wound infection	0		1		
Recollapse	12		2		
Refracture	2		0		
Operation time (mins)	46.8 ± 9.7		81.7 ± 12.7		0.02^∗^
Blood loss (ml)	8.6 ± 2.1		74.7 ± 9.3		0.01^∗^

^∗^ *p* < 0.05; *p*_1_: the comparison between two groups; *p*_2_: the comparison with last follow-up; VAS: visual analogue scale; ODI: Oswestry Disability Index.

**Table 3:** Comparison of AVHr and CA between the two groups.

Variable	PKP group (*n* = 47)		PKPF group (*n* = 40)		*p*_1_
AVHr (%)		*p*_2_		*p*_2_	
Preoperative	48.9 ± 9.1		47.3 ± 11.3		0.73
1 month	92.1 ± 2.6	<0.001^∗^	95.7 ± 3.1	<0.001^∗^	0.02^∗^
6 months	91.3 ± 1.5	0.24	93.2 ± 2.8	0.15	0.03^∗^
12 months	81.7 ± 3.2	0.04^∗^	92.7 ± 2.3	0.67	0.01^∗^
24 months	80.1 ± 3.2	0.36	92.8 ± 2.5	0.82	0.02^∗^
CA (degree)					
Preoperative	20.5 ± 2.5		21.1 ± 3.1		0.57
1 month	6.4 ± 3.1	<0.001^∗^	3.1 ± 2.5	<0.001^∗^	0.02^∗^
6 months	9.7 ± 4.5	0.03^∗^	3.4 ± 2.1	0.23	0.01^∗^
12 months	9.6 ± 2.5	0.72	3.8 ± 1.8	0.42	0.03^∗^
24 months	10.5 ± 3.1	0.82	4.5 ± 1.5	0.38	0.02^∗^

^∗^ *p* < 0.05; *p*_1_: the comparison between two groups; *p*_2_: the comparison with last follow-up; AVHr: anterior vertebra height; CA: Cobb angle.

## Data Availability

Data is available via a request to the corresponding author.
